# The β-amyloid peptide compromises Reelin signaling in Alzheimer’s disease

**DOI:** 10.1038/srep31646

**Published:** 2016-08-17

**Authors:** Inmaculada Cuchillo-Ibañez, Trinidad Mata-Balaguer, Valeria Balmaceda, Juan José Arranz, Johannes Nimpf, Javier Sáez-Valero

**Affiliations:** 1 Instituto de Neurociencias de Alicante, Universidad Miguel Hernández-CSIC, Sant Joan d’Alacant, E-03550, Spain; 2 Centro de Investigación Biomédica en Red sobre Enfermedades Neurodegenerativas (CIBERNED), Spain; 3 Departamento Producción Animal, Universidad de León, León, Spain; 4 Department of Medical Biochemistry, Max F. Perutz Laboratories, Medical University of Vienna, 1030 Vienna, Austria

## Abstract

Reelin is a signaling protein that plays a crucial role in synaptic function, which expression is influenced by β-amyloid (Aβ). We show that Reelin and Aβ oligomers co-immunoprecipitated in human brain extracts and were present in the same size-exclusion chromatography fractions. Aβ treatment of cells led to increase expression of Reelin, but secreted Reelin results trapped together with Aβ aggregates. In frontal cortex extracts an increase in Reelin mRNA, and in soluble and insoluble (guanidine-extractable) Reelin protein, was associated with late Braak stages of Alzheimer’s disease (AD), while expression of its receptor, ApoER2, did not change. However, Reelin-dependent induction of Dab1 phosphorylation appeared reduced in AD. In cells, Aβ reduced the capacity of Reelin to induce internalization of biotinylated ApoER2 and ApoER2 processing. Soluble proteolytic fragments of ApoER2 generated after Reelin binding can be detected in cerebrospinal fluid (CSF). Quantification of these soluble fragments in CSF could be a tool to evaluate the efficiency of Reelin signaling in the brain. These CSF-ApoER2 fragments correlated with Reelin levels only in control subjects, not in AD, where these fragments diminished. We conclude that while Reelin expression is enhanced in the Alzheimer’s brain, the interaction of Reelin with Aβ hinders its biological activity.

Reelin is a large glycoprotein implicated in the regulation of synaptic neurotransmission, plasticity and memory in the adult brain^1^. Reelin signals through the apolipoprotein E receptor 2 (ApoER2) or the very-low-density liporeceptor (VLDLR)^2^,^3^, both of which also bind apolipoprotein E (ApoE). Significantly, the ApoE4 variant is the largest known genetic risk factor for late-onset sporadic Alzheimer’s disease (AD)^4^. Reelin binding induces the cleavage of ApoER2 through the sequential processing of α- and γ-secretases, enzymes that also process the β-amyloid precursor protein, APP^5^,^6^. Reelin relays an intracellular signal via the Dab1 adapter (Disabled-1), triggering an intracellular kinase cascade that ultimately inhibits glycogen synthase kinase-3β (GSK3β) and prevents tau hyperphosphorylation^7^.

A growing number of studies have demonstrated interactions between β-amyloid peptide (Aβ) and Reelin, or the elements in its signaling pathway. Both Reelin and Dab1 have been shown to interact with APP^8^, and Reelin influences its trafficking and processing^9^,^10^. Reelin is also able to antagonize the suppression of synaptic transmission exerted by Aβ^11^. In addition, Reelin co-localizes with oligomeric Aβ aggregates in the hippocampus of aged mice^12^, and a direct interaction between Reelin and β-amyloid has also been demonstrated *in vitro*^13^. These links between Reelin signaling and Aβ have augmented the interest in the implication of Reelin in AD. However, there is contradictory data regarding the amount of Reelin in the brain of AD patients and in mice over-expressing Aβ, reporting both, depletion^8^,^14^,^15^ and increase^16^,^17^,^18^ of Reelin in both settings. We previously demonstrated that Aβ triggers an increase in Reelin^17^, while it also compromises Reelin function, probably by modifying its glycosylation^19^. These findings support the notion that Aβ influences Reelin expression and that it compromises its biological activity, although signaling through Reelin has not yet been explored in AD.

## Results

### Aβ triggers increased Reelin expression and interacts with Reelin in the human brain

**Figure 1:**
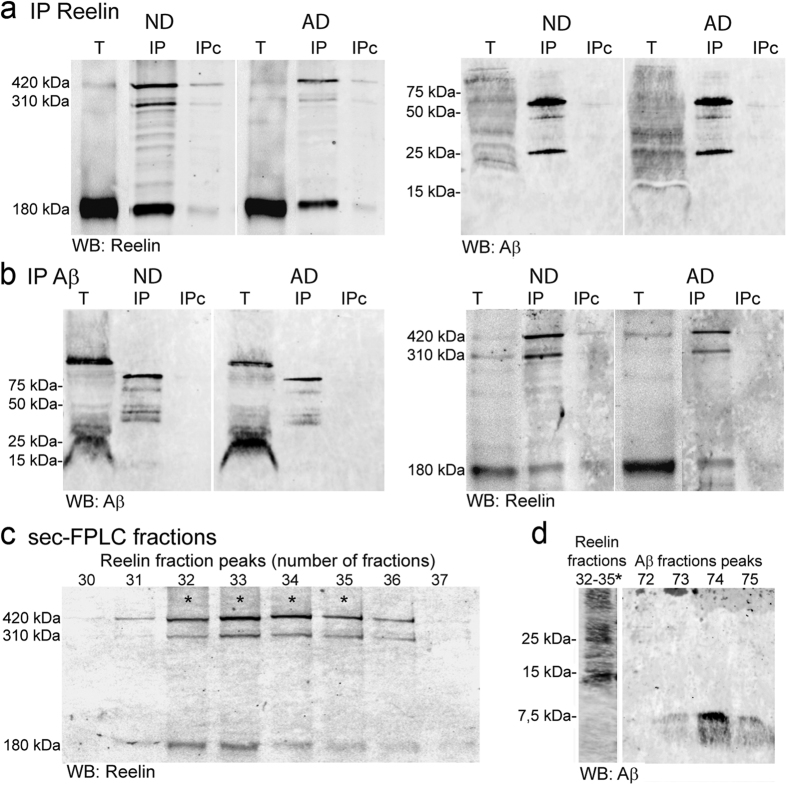
Reelin and Aβ interact in human brain tissue. Immunoprecipitation of human frontal cortex extracts, non-disease control (ND) and AD with (**a**) anti-Reelin (G10+CR50), or (**b**) anti-Aβ (4G8) antibodies (n = 3; T, total lysate). The immunoprecipitated proteins (IP) were probed with the 142 antibody against Reelin and the 6E10 antibody against Aβ. The Reelin antibody detects the 420 kDa full-length and the 310 and 180-kDa fragments. Extracts incubated with beads in the absence of antibody were negative controls (IPc). (**c**) Reelin blot from cortex extracts fractioned by size-exclusion. (**d**) Reelin-rich fractions were also pooled together and probed with the 4G8 antibody against Aβ, and analyzed for comparison with fractions containing small Aβ oligomers. Note that Reelin co-immunoprecipitates and interacts mostly with oligomeric Aβ species. One AD case is shown but similar elution profiles were obtained for ND cases.

We examined the possible interaction between Reelin and Aβ by immunoprecipitating Reelin from human frontal cortex extracts from non-demented/non-disease (ND) and AD subjects using a combination of the G10 and CR50 antibodies that recognize the N-terminal region of the protein. Immunoprecipitation of the full-length Reelin (~420 kDa) and the ~310 kDa and ~180 kDa Reelin fragments was confirmed in Western blots probed with the anti-N-terminal Reelin 142 antibody. Considerable amounts of oligomeric Aβ species were detected by the 6E10 antibody in the Reelin immunoprecipitates (Fig. 1a); protein bands that were not observed in the absence of antibody. To confirm this interaction, we performed reverse co-immunoprecipitations with the 4G8 antibody that could pulls down monomeric and oligomeric Aβ species. Western blots of the immunoprecipitates probed with the anti-Reelin antibody corroborated the interaction between Reelin and Aβ (Fig. 1b), and no Reelin was co-immunoprecipitated in the absence of the antibody against Aβ. To further demonstrate the association between Reelin and Aβ, we purified Reelin from extracts of the ND and AD cortex by size-exclusion chromatography. Aβ oligomers were clearly detected when the fractions rich in full-length Reelin were pooled and probed with the 6E10 antibody (Fig. 1c), suggesting that Aβ interacts with high molecular weight proteins including Reelin (Fig. 1d).

**Figure 2:**
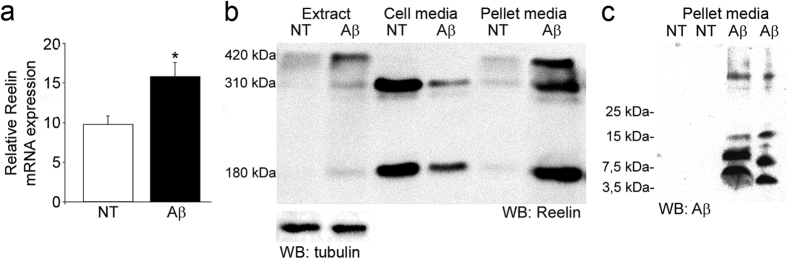
Aβ42 increases Reelin expression in cell culture and is trapped into Aβ fibers. (**a**) Differentiated SH-SY5Y cells were treated with 10 μM of Aβ42 and mRNA Reelin was determined and compared with non-treated (NT) cells. Data represents relative Reelin mRNA levels normalized to GAPDH and are expressed as mean values ± SEM of 10 independent determinations from at least 2 different experiments (*p < 0.001). (**b**) Western blots of SH-SY5Y cell extracts, the media and the pellet from the media of cells maintained in the presence or absence of Aβ42, and probed for Reelin (representative blot, n = 3) and α-tubulin from cell extracts as a loading control. (**c**) Proteins recovered in the pellet from the media were also probed with the 4G8 antibody against Aβ.

We also tested the effect of Aβ42 on differentiated SH-SY5Y cells that secrete Reelin. Remarkably, the mRNA Reelin content was increased in cellular extracts treated with the peptide (Fig. 2a). Accordingly, exposing these cells to Aβ42 augmented the cellular Reelin protein levels relative to untreated cells, while reduced amount of secreted Reelin was detectable into the culture media (Fig. 2b). A previous study indicated that increased Reelin levels in cell treatment with Aβ42 did not result from altered secretory pathway^17^. When the material pelleted by centrifugation of the culture media was examined, we found a large amount of Reelin together with Aβ oligomers almost exclusively in Aβ-treated cells (Fig. 2c), indicating that a notable amount of secreted Reelin could be associated with the Aβ fibers formed during the 4 day exposure to the peptide. Since Aβ42 exposure induced a degree of death cell (22 ± 3%, MTT reduction; *p* < 0.001), some of the Reelin recovered from the media may originate from these dead cells.

### Reelin expression increases in the brain of AD patients

**Figure 3:**
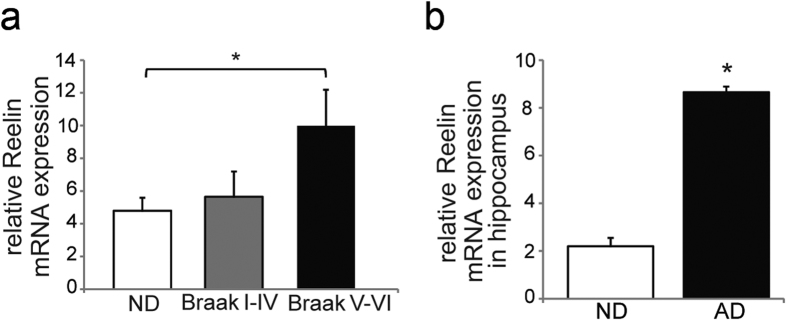
Increased Reelin expression in the brain at advanced Braak stages of AD. AD subjects were categorized as early (I-II to IV) or advanced Braak stages (V to VI). (**a**) Relative Reelin mRNA expression analyzed by *q*RT-PCR in frontal cortex samples from ND (n = 11) and AD subjects (stages I to IV, n = 7; stages V to VI, n = 10). (**b**) Reelin mRNA was also analyzed in the hippocampus of ND controls (ND; n = 5) and AD samples corresponding to Braak stages IV to VI (n = 9). The values were calculated from relative standard curves and normalized to GAPDH from the same cDNA preparation, confirming the specificity of the PCR products from dissociation curves. The data represent the means ± SEM. *p < 0.05 using Student’s *t*-test.

Reelin expression in the frontal cortex of AD and ND was evaluated by *q*RT-PCR. When AD cases were classified with respect to their Braak and Braak stage, we found a two-fold increase in relative Reelin mRNA expression in extracts from late AD Braak stages (V to VI; *p* = 0.03) with respect to ND (Fig. 3a). In contrast, no differences were observed at Braak stages I–II to V. We also determined the Reelin expression in the hippocampus by *q*RT-PCR at Braak stages IV to VI (the only tissue available from our collection) and again, Reelin mRNA was expressed more strongly in the AD hippocampus at these stages that in the ND tissue (*p* = 0.02; Fig. 3b).

**Figure 4:**
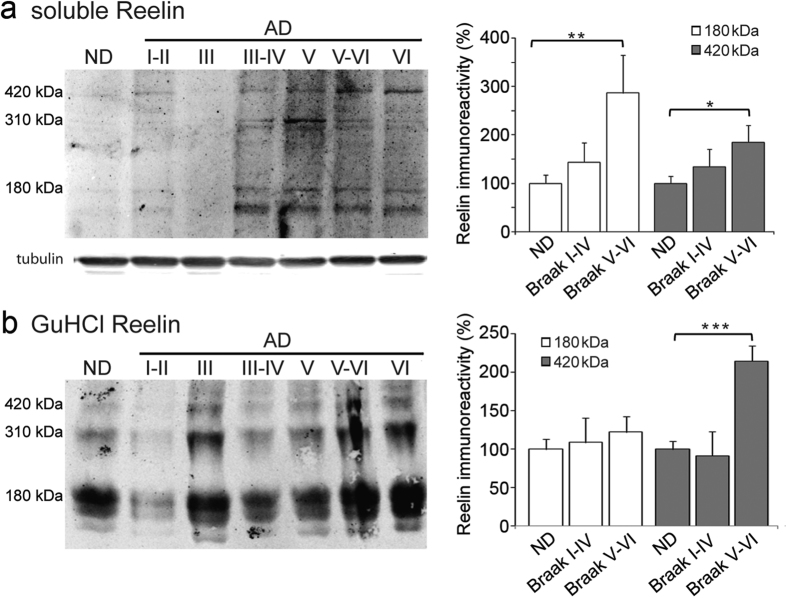
An increase in Reelin protein in the frontal cortex at advanced Braak stages of AD. Frontal cortex samples from ND (n = 11) and AD subjects (n=17) were homogenized in detergents diluted in Tris-saline buffer (soluble Reelin, (**a**) and the pellets recovered were re-extracted in Guanidine-HCl (GuHCl Reelin, (**b**). Western blots were probed with the 142 antibody (α-tubulin served as a loading control). AD subjects were categorized as early (I–II to IV; n = 7) or advanced Braak stages (V to VI; n = 10). The data represent the means ± SEM and were normalized with respect to the ND values. The data represent the means ± SEM. *p < 0.05, **p < 0.01, ***p < 0.001, using Student’s *t* test.

We also compared the levels of soluble full-length Reelin in Western blots of AD and ND brain extracts. We found a significant increase in full-length Reelin in extracts from advanced stages of AD (stages V to VI, 85% increase; *p* = 0.02) with respect to the ND extracts (Fig. 4a). Similarly, an increase in the major 180 kDa Reelin fragment was also evident at these stages with respect to ND (186% increase; *p* = 0.003). By contrast, no significant differences were found between extracts from earlier Braak stages (I–II to IV) and ND extracts.

Since previous cell culture experiments suggested that Reelin could be sequestered into Aβ fibers, we also analyzed the levels of Reelin in amyloid pellets. Brain tissue pellets were re-suspended and solubilized with GuHCl to extract the insoluble amyloid from AD brains^20^, and when the GuHCl extractable full-length Reelin was quantified (Fig. 4b) the same tendency as that seen for soluble Reelin was observed. Thus, there was significantly more full-length Reelin in AD samples from Braak stages V to VI (114% increase; *p* = 0.001) than in ND extracts but not from earlier Braak stages (I–II to IV).

Handling could influence the stability and measurement of Reelin, particularly its denaturation during sample preparation for electrophoresis^16^,^21^. Since Reelin isolated from the AD brain exhibits a different glycosylation pattern in comparison to Reelin from ND extracts^16^,^17^, sample handling could affect different Reelin glycoforms to a different extent. In fact, a 5 min boiling of the sample prior to SDS-PAGE apparently diminished the Reelin in the AD cortex by up to 70% compared to that detected after 3 min at 98 °C, whereas the Reelin in the cortex from ND cases was less strongly affected by 5 min boiling, diminishing it only for ~25% with respect to 3 min denaturation. These data suggested that Reelin from AD extracts is particularly sensitive to temperature, and that the differences between the Reelin in AD and ND extracts could be influenced by temperature (see Supplementary Fig. 1). In this study, a 3 min denaturation at 98 °C was used to study Reelin in Western blots.

In order to confirm the results from the Western blots, brain Reelin was also quantified using an ELISA specific for human Reelin (USCN, Life Science Inc) that does not involve sample denaturation by heating. To our knowledge, this kit has not been validated previously and thus, we first confirmed that this ELISA could detect full-length and N-terminal fragments of brain Reelin by analyzing the unbound fraction in Western blots. When fresh aliquots of AD and ND brain extracts were analyzed Reelin protein was increased ~97% (*p* = 0,043) in the entire AD group (0.18 ± 0.03 ng/ml) with respect to that in the ND subjects (0.09 ± 0.01 ng/ml).

### ApoER2 expression does not change but Dab1 is less phosphorylated in AD brain

**Figure 5:**
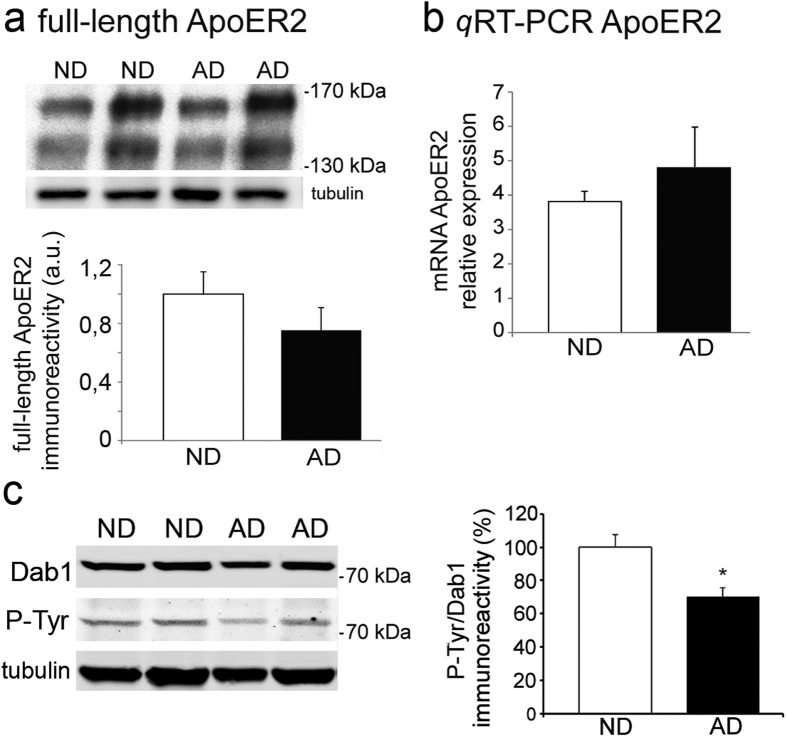
ApoER2 levels remain unaltered in the AD frontal cortex but Dab1 phosphorylation decreases. (**a**) Quantification of ApoER2 (~130 kDa + ~170 kDa) in the ND (n = 10) and AD cortex (n = 12) in Western blots probed with a C-terminal ApoER2 antibody and α-tubulin as loading control. (**b**) Relative ApoER2 mRNA expression analyzed by *q*RT-PCR in ND (n = 8) and AD (n = 11) frontal cortex, calculated from standard curves and normalized to GAPDH. Data represent the mean ± SEM. None of the comparisons resulted in significant differences, considering the entire AD group or the advanced Braak stages. (**c**) Western blots of frontal cortex extracts from ND (n = 12) and AD (n = 15) revealed by fluorescence simultaneously for an anti-Dab1 antibody and an anti-phosphotyrosine (P-Tyr), and α-tubulin as loading control. Data represent the means ± SEM and were normalized with respect to the ND values. *p < 0.005 using a Student’s *t* test.

Levels of ApoER2 were analyzed in ND and AD brain extracts. There were no significant differences in the amount of full-length ApoER2 protein, quantified by Western blots (Fig. 5a), neither in the ApoER2 mRNA quantified by *q*RT-PCR (Fig. 5b) between AD, for any Braak stage, and ND subjects.

Phosphorylation of the downstream protein Dab1 was also examined. Quantitative analyses showed a decrease (30%; *p* = 0,01) in tyrosine phosphorylation of Dab1 in the entire AD group with respect to that in the ND group (Fig. 5c). A different combination of P-Dab1/Dab1 antibodies served to ensure the identity of the immunoreactive bands and confirm the decrease (36%; *p* = 0,004) in tyrosine phosphorylation of Dab1 (Supplementary Fig. 2).

### Aβ impairs Reelin signaling

**Figure 6:**
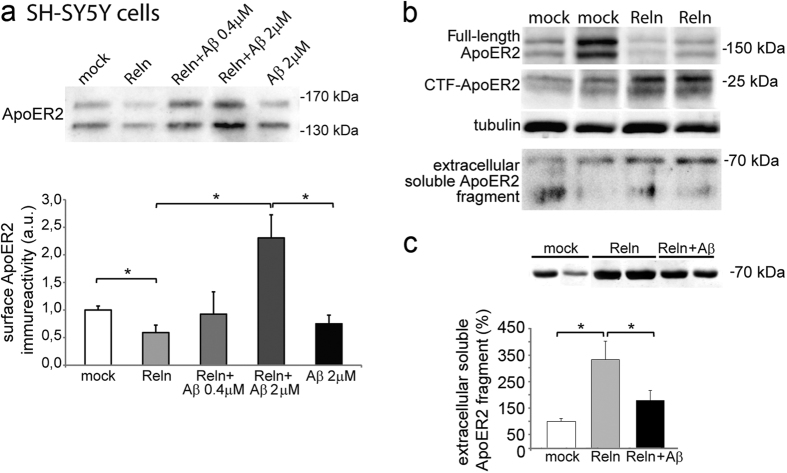
Aβ impairs the binding of Reelin to ApoER2. (**a**) Aβ increases the presence of ApoER2 at the cytoplasmic membrane induced by Reelin. Quantification of biotinylated ApoER2 (~130 kDa + ~170 kDa) from untreated SH-SY5Y cells and those treated with Reelin alone (Reln; ~10 nM), with Aβ42 or with Reelin previously incubated with Aβ42 at the concentration indicated. Note that Aβ42 (2 μM) alone did not have noticeable effect. Data from 6 independent experiments are normalized with respect to the mock values and represented as the means ± SEM: *significantly different (*p* < 0.05) using a Student’s *t* test. (**b**) Reelin binding induces cleavage of the ApoER2 receptor in SH-SY5Y cells, generating a soluble ApoER2 fragment (~70 kDa) in the culture medium of treated cells that can be monitored with the 186 antibody against ApoER2. The processing of the full-length ApoER2 receptor was also assessed by the appearance of an intracellular C-terminal fragment (CTF). (**c**) The presence of Aβ (2 μM) weakens generation of the soluble 70 kDa ApoER2 fragments. Data represent the means ± SEM normalized with respect to the mock values (8 determinations from 2 separate experiments). *p < 0.005 using Mann-Whitney test.

The effect of Aβ42 on Reelin signaling was tested by analyzing the fate of ApoER2 in protein-surface biotinylated SH-SY5Y cells. Cells overexpressing full-length ApoER2-Cherry were stimulated with recombinant Reelin (~10 nM), Aβ42 or with Reelin (~10 nM) plus Aβ42 previously incubated together. After 15 min at 37 °C, the surface biotinylated proteins were pulled down and probed in Western blots with an antibody against ApoER2 (Fig. 6a). Reelin reduced the presence of ApoER2 at the cell membrane with respect to the controls, but when cells were treated with Reelin and 2 μM Aβ42, plasmatic membrane ApoER2 exposition increased noticeably (*p* = 0.04).

Reelin binding induces the clustering and the proteolytic processing of ApoER2, generating a soluble extracellular fragment^22^,^23^, that can be recognized by the 186 antibody raised against the entire ligand binding domain of the receptor^24^. The generation of this extracellular fragment could be observed in SH-SY5Y cells over-expressing ApoER2 after treatment with Reelin, which induces the appearance of a ~70 kDa ApoER2 fragment in the culture medium (Fig. 6b). In contrast, a lower amount of this soluble fragment was measured in the medium of cells treated with Reelin plus Aβ42 (Fig. 6c).

Previously we demonstrated that Reelin obtained from SH-SY5Y cells treated with Aβ42 fails to reduce tau phosphorylation^19^. Here we tested if Aβ directly impairs the effect of Reelin in the modulation of tau phosphorylation. Neurons treated with Reelin in presence of Aβ42 fail to induce phosphorylation of Dab1 and to reduce tau phosphorylation (see Supplementary Fig. 3). These results confirm that Aβ compromises the biological role of Reelin modulating tau phosphorylation.

### Evidences for a Reelin signaling impairment in AD

**Figure 7:**
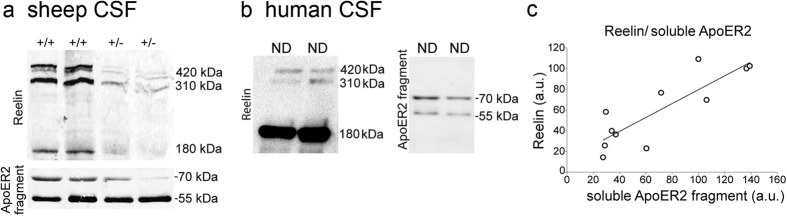
Characterization of the soluble ApoER2 fragment present in CSF. (**a**) Western blots of Reelin and soluble ApoER2 fragments of CSF from homozygous (+/+) and heterozygous (+/−) sheep carrying a mutation that reduces Reelin expression (representative blot, n = 2). (**b**) Western blot showing the presence of Reelin and soluble ApoER2 fragments in human CSF, and (**c**) the correlation between full-length Reelin and the 70-kDa soluble ApoER2 fragment in human non-disease (ND; n = 11 from different ages) CSF samples.

Following our interest to study the malfunctioning of Reelin signaling in the AD brain, we postulated that quantifying the soluble ApoER2 fragments in the CSF would reflect the efficiency of Reelin signaling, given that we have not found traces of full-length ApoER2 in cerebrospinal fluid (CSF). Indeed, we found a reduction of the ~70 band detected by the 186 antibody in the CSF from a heterozygous Reelin mutant sheep^25^ (Fig. 7a). Likewise, the 186 antibody detected similar ApoER2 fragments in human CSF from ND cases (Fig. 7b), with the most abundant 70 kDa ApoER2 fragment positively correlated with the full-length Reelin levels (r = 0.85, *p* < 0.001; Fig. 7c).

**Figure 8:**
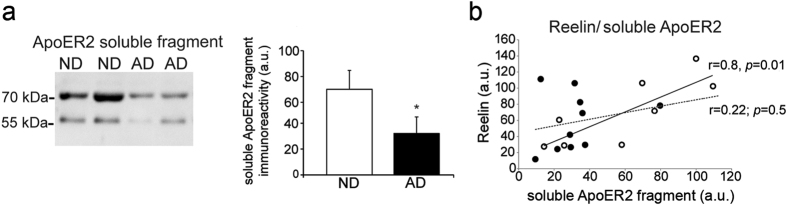
Decrease of the soluble ApoER2 fragment in AD CSF. (**a**) Samples from age-matched ND controls (n = 8) and AD patients (n = 10) were assayed for Reelin by immnoblotting and ELISA and selected on the basis of a similar Reelin content (comparison is shown). (**b**) Representative blots and densitometric quantification of the 70 kDa soluble ApoER2 fragment assayed with the 186 antibody in samples. (**c**) Correlation between the soluble full-length Reelin and ApoER2 soluble fragment in ND (open circles) and AD (closed circles) CSF samples. The dashed line represents the lack of correlation in AD samples.

The soluble ApoER2 fragment was measured in the CSF from AD and age-matched ND subjects. The CSF samples were selected on the basis of a similar Reelin content (Fig. 8a); Reelin from AD CSF also displayed the characteristic altered glycosylation pattern of Reelin from ND samples^17^. In the CSF from AD subjects there was a significant decrease (~54%) in the 70 kDa soluble ApoER2 fragment, with respect to that from ND subjects (Fig. 8b). Moreover, the ApoER2 fragments were not correlated with the amount of Reelin in AD samples, suggesting a disparity between Reelin and ApoER2 in AD (Fig. 8c).

Altogether, these results indicate that the Reelin present in the AD brain is targeted by Aβ. The association between these proteins hinders the processing of ApoER2 and probably Reelin signaling in AD.

## Discussion

A “functional” link between Reelin and Aβ has previously been demonstrated, whereby Reelin delays Aβ fibril formation *in vitro*^13^ and it protects against toxicity *in vivo*^13^,^26^. In anatomical studies, Reelin has been found associated with Aβ in transgenic mice model of AD and in aged wild-type mice^12^,^27^,^28^. However, the interaction between Reelin and Aβ in human AD brains had not been explored so far. In this study, we present evidence that soluble Reelin interacts with Aβ in ND and AD human brain homogenates. This interaction elicits an increase in Reelin expression in cells, but decreases the internalization of ApoER2 from the cytoplasmic membrane; thus Aβ hinders Reelin biological activity and ultimately could influence pathological progression of Alzheimer’s disease by impairing Reelin signaling.

In frontal cortex tissue from advanced stage AD patients (Braak stage V to VI), we found a significant increase in soluble and GuHCl extractable Reelin. These advanced Braak stages correspond to the phase of neocortical lesion expansion into high order association areas, such as the frontal, parietal and occipital neocortex^29^. Increased Reelin expression was also demonstrated by *q*RT-PCR in the frontal cortex at late Braak stages, as confirmed in the hippocampus, another brain area affected in AD. An up-regulation of Reelin transcripts in the frontal cortex of AD patients has been proposed previously^16^,^17^,^30^. Moreover, large increases in Reelin protein and mRNA have also been described in the brain of individuals with Down syndrome, where APP is overexpressed^17^. Thus, the dynamics of the Reelin and Aβ interaction, and the associated changes provoked in Reelin expression, appear to be related to disease progression, a phenomenon that merits further investigations. We presume that oligomeric Aβ is the specie that triggers the increase in Reelin expression; however, under the conditions of our cellular experiments, we cannot differentiate if this effect is due to oligomeric Aβ or fibers. Anyhow, Aβ fibers might have also a role in compromising Reelin activity since Reelin results trapped into Aβ aggregates.

There are currently some discrepancies regarding the levels of Reelin in the brain. In some studies Reelin depletion has been described in AD brains^14^,^15^, whereas an increase in Reelin has also been observed^16^,^17^,^18^. These differences could be due to the reduced sample size used in some studies, while factors related to the handling of samples also emerge as a potentially important cause to influence the evaluation of Reelin protein (discussed in ref. 31). Here, we demonstrated that heating affects the detection of Reelin in protein extracted from AD brains, which displays differences in glycosylation. It is well known that changes in glycosylation can influence protein stability and limit a protein’s half-life in circulation^32^,^33^. However, the ELISA assay used here, which allows protein content to be determined without heating the samples, confirmed that soluble Reelin levels are higher in the AD cortex.

A relevant question that still remains is whether Reelin is biologically active in the AD brain and consequently, whether the integrity of Reelin signaling is retained. Previous studies described that the levels of the intracellular adapter Dab1 mRNA are upregulated^34^ or unaffected^18^ in frontal cortex of AD patients, although the study of Dab1 phosphorylation has not been addressed yet. Our data indicate an abnormal Reelin signaling in the brain of AD patients, since levels of phosphorylated Dab1 were decreased respect to total Dab1. Moreover, we demonstrate that Aβ can directly interfere with the Reelin-dependent internalization of its receptor, ApoER2. ApoER2 appears to be the dominant Reelin receptor in the human forebrain, participating in the modulation of synaptic plasticity and memory formation^35^,^36^. We examined the presence of a proteolytic ApoER2 fragment in the CSF, generated by cleavage of the receptor after Reelin binding. We quantified this soluble ApoER2 fragment in CSF as a read-out of Reelin signaling, which appears to be a better indicator than Reelin fragments. Although Reelin processing is derived to some extent from its interaction with the receptor^37^,^38^,^39^, fragments of Reelin can also be generated through the activity of extracellular matrix metalloproteinases^40^,^41^,^42^,^43^, independent of ApoER2, ruling out the quantification of Reelin fragments as a suitable read-out of its signaling function. In CSF from ND subjects, the levels of ApoER2 fragments correlate with that of full-length Reelin, while in AD subjects this correlation does not exist and the levels of the soluble ApoER2 fragment decreases, even though ApoER2 expression did not change. Therefore, while there is more Reelin in the AD brain, less soluble ApoER2 fragments are found in the CSF. As such, quantifying the circulating ApoER2 fragments may be useful to assess the effects on Reelin signaling in other neuropsychiatric disorders where the expression of Reelin is altered^44^. In this context, to assay levels of ApoER2 transcripts in brain of AD subjects we designed primers common for all splicing variants. Recent finding indicate that the balance of ApoER2 splicing variant is deregulated in brain from AD patients and in a transgenic mouse model of AD^45^. Indeed, differential splicing and glycosylation of ApoER2 regulates its role in synaptic function and memory^46^. Further studies should clarify the role of deregulated ApoER2 splicing in AD.

Cleavage of ApoER2 occurs through sequential α- and γ-secretase processing, resulting in the release of a soluble intracellular domain (ICD)^5^,^23^. We recently reported that this ApoER2-ICD down-regulates *RELN* promoter activity and consequently Reelin expression^47^. While there is an increase in Reelin in the AD cortex, this Reelin is glycosylated distinctly to that in the ND cortex^17^. The altered Reelin glycosylation induced by Aβ appears to impair its efficient binding to ApoER2, dampening the down-regulation of tau phosphorylation via the GSK3β kinase^19^. Thus, Aβ could establish a vicious circle in the pathological condition, whereby a less-functional Reelin would generate fewer ApoER2-ICD fragments that would in turn increase Reelin transcription, as occurs in the AD brain. Therefore, the effect of Aβ on Reelin in the AD brain might induce chronic signaling failure, which would consequently affect synaptic neurotransmission, plasticity and memory.

Finally, the most robust evidence that links impaired Reelin-ApoER2 signaling with AD neurodegeneration might be the increase in tau phosphorylation. Indeed, tau phosphorylation and fibrillary tangles are more closely associated with the severity of memory loss in humans than Aβ^48^,^49^,^50^. Perhaps the influence of Aβ in the impaired Reelin brain function reveals a cross-talk between disturbed tau phosphorylation and β-amyloid. Previous data demonstrated that Reelin forms induced by β-amyloid are less capable of down-regulating tau phosphorylation via Dab1 and GSK3β^19^. Our data associates Aβ and tau phosphorylation dysregulation through Reelin and raises the possibility that Reelin directly contributes to the progress of AD pathology. The mechanism of mutual influencing and the role of Reelin deserve attention.

## Methods

### Collection of human brain and CSF samples

This study was approved by the ethic committee of Universidad Miguel Hernández de Elche, Spain, and it was carried out in accordance with the Helsinki Declaration. Brain samples (frontal cortex and hippocampus) were obtained from the UIPA neurological tissue bank (Unidad de Investigación Proyecto Alzheimer; Madrid), in which sporadic AD cases [n = 17 (9 female/8 male); 83 ± 1 years] were categorized according to the Braak and Braak stage^29^. Samples from ND individuals (n = 11 (4 female/7 male); 63 ± 3 years) corresponded to individuals with no clinical dementia and no evidence of a brain pathology. The mean post-mortem interval of the tissue was 6 h in all cases, with no significant difference between the groups. CSF samples from probable AD cases (n = 10; 77 ± 2 years) and ND controls (n = 8; 72 ± 3 years) were collected at the “Hospital Clínico San Carlos”, Madrid, Spain. All experiments were carried out in accordance with the Helsinki Declaration guidelines and regulations. All experimental protocols were approved by ethic committee of Hospital Clínico San Carlos and Universidad Miguel Hernández de Elche. An informed consent was obtained from all subjects. All AD patients fulfilled the NINCDS-ADRDA criteria for “probable” AD^51^.

CSF samples were also collected from two 5-year-old sheep heterozygous for a naturally-occurring Reelin mutation and two age-matched controls. The mutants carried a 31 bp deletion in the *RELN* gene that produces a premature termination codon and the loss of the Reelin protein^25^. Sheep were sampled by cerebellomedullary cistern puncture at the Veterinary Faculty of Leon, Spain, by qualified veterinarians following standard procedures^52^ and conducted under license issued in accordance with European Union legislation (European Community Directive, 86/609/EC and Directive 2010/63/EU of the European Parliament and of the Council). Experimental protocols were approved by the ethic committee of Universidad de Leon, Spain. All animals were managed in accordance with the guidelines for the accommodation and care of animals.

### Reelin and Aβ solubilization from human brain samples

Tissue from the frontal cortex (~0.1 g) was homogenized in solubilization buffer (10% w/v): Tris-HCl (50 mM, pH 7.4), NaCl (150 mM), Triton X-100 (0.5%), Nonidet P-40 (0.5%) and a cocktail of protease inhibitors^17^. The homogenates obtained were sonicated, centrifuged for 20 min at 20,000×*g* and 4 °C, and the supernatants were recovered. To release the insoluble proteins aggregated to Aβ, 10 volumes of 5 M Guanidine-HCl (GuHCl) diluted in Tris HCl (20 mM, pH 8.0) was added to the pellets, and the solution was then diluted 1:10 in PBS with BSA (0.5%), Tween-20 (0.05%) and protease inhibitors. After centrifugation for 25 min at 13,100×*g* and 4 °C, the supernatants were again recovered.

Aβ immunoprecipitation assays were performed on human frontal cortex samples that were homogenized (25% w/v) in Tris-HCl (20 mM, pH 7.4), NaCl (150 mM), TX-100 (1% w/v), EGTA (1 mM), EDTA (5 mM), NaF (50 mM), NaVO_3_ (0.1 mM) and protease inhibitors. After 25 strokes with a homogenizer, the lysates were centrifuged for 30 min at 175,000×*g* and 4 °C and the supernatants recovered.

### Production of recombinant Reelin

HEK-293T cells stably transfected with Reelin and GFP (mock) (kindly provided by Dr. E. Soriano, Department of Cell Biology, University of Barcelona, Barcelona, Spain) were seeded in 175 cm^2^ flasks at a density of 10 × 10^6^ cells/flask. After 3 days in culture in Optimem, the supernatants were filtrated through 0.2 μm pore and concentrated with an Amicon Ultra 100 kDa size exclusion filter (Merk Millipore, Darmstadt, Germany). To calculate the Reelin concentration, several dilutions of Reelin were compared to known concentrations of albumin in a Coomassie gel.

### Cell cultures and Aβ treatment

SH-SY5Y cells were differentiated with all-trans-retinoic acid (Sigma) and brain-derived neurotrophic factor (BDNF, Sigma Aldrich, MO, USA), and suspensions of β-amyloid 1–42 peptide (Aβ42, final concentration of 10 μM: American Peptide Company Inc.) were added every other day over 4 consecutive days. Subsequently, the medium was removed, and the cells were suspended in 100 μL of solubilization buffer and processed as described above. Cells cultured in 96-well plates and treated as described above were assessed for viability using the tetrazolium assay (MTS: CellTiter 96^®^ AQueous Assay, Promega, Southampton, UK).

The primary cortical neuron cultures were performed as in Cuchillo-Ibanez *et al.*^19^. Briefly, neurons from cortical lobes of E16.5 mice embryos were plated onto 35-mm dishes (1.3 × 10^6^ cells/dish) and maintained in Neurobasal medium (Invitrogen) containing B27 supplements (GIBCO BRL), 100 IU/ml penicillin, 100 μg/ml streptomycin and 2 mM glutamine. After 7 days, cortical neurons were treated for 45 min with Neurobasal medium (mock), ~10 nM Reelin or 10 nM Reelin together with 2 μM synthetic Aβ42 (American Peptide Company Inc, Vista, CA, USA).

### Western Blotting and antibodies

For western blotting, CSF samples (30 μL), brain extracts (30 μg), neuronal extracts (40 μg), SH-SY5Y extracts (25 μL) and SH-SY5Y supernatants (25 μL) were boiled for 3 (Reelin detection) or 5 min (ApoER2, Dab1 and tau detection) at 98 °C, or for 15 min at 65 °C for Aβ. After resolving by SDS-PAGE, the proteins were transferred to membranes and detected with antibodies against Reelin (clone142, Merck Millipore), ApoER2 (clone 186 for the soluble fragment including ligand domain^24^ or an antibody against the C-terminal for the full-length protein, Abcam), Dab1 (goat polyclonal, Abcam), Dab1 (rabbit polyclonal Merk Millipore), anti-phospho Y232 Dab1 (rabbit monoclonal, Abcam), 4G10 Platinum anti-phosphotyrosine antibody (mouse monoclonal, Merck Millipore), tau (DakoCytomation), anti-phospho PHF tau P-Thr212/Ser214 (AT100, Thermo Scientific) and α-tubulin (Sigma-Aldrich) as a loading control. Aβ peptides were resolved by 16% Tris-tricine SDS-PAGE and detected with the 6E10 antibody (Covance Research). Antibody binding was visualized with fluorescent secondary IRDye antibodies and recorded on an Odyssey CLx Infrared Imaging system (LI-COR Biosciences GmbH).

### Lectin binding analysis of Reelin

Aliquots of CSF (100 μL) were mixed with 40 μL of immobilized lectins (*Canavalia ensiformis*, Con A or *Lens culinaris agglutinin* -LCA: Sigma-Aldrich). After overnight incubation at 4 °C, unbound Reelin was separated by centrifugation and examined in Western Blots.

### Reelin and Aβ immunoprecipitation from the human cortex

Brain extracts (200 μL) were incubated for 2.5 h at room temperature (on a roller) with 100 μL Dynabeads (Merk Millipore) coupled to either the CR50 (Covance) and G10 (Millipore) antibodies against Reelin or the 4G8 antibody against Aβ (Covance).

### Size-exclusion chromatography

Aliquots of brain extracts (1 mL) were injected onto a Superdex 200 HiLoad 16/60 column using an ÄKTA-Prime FPLC system (GE Healthcare Life Sciences, Barcelona, Spain) and 1.4 mL fractions were eluted at a flow rate of 0.6 mL/min using NaH_2_PO_4_ (50 mM, pH 7), NaCl (150 mM).

### ELISA for Reelin

Brain samples were assayed (30 μg/well) using the commercial SEC775Hu ELISA kit (USCN Life Science Inc., Wuhan, P.R. China) according to the manufacturer’s instructions.

### Biotinylization assay

SH-SY5Y cells overexpressing full-length ApoER2-Cherry for 24 h were incubated with supernatant media from GFP-HEK cells (mock), ~10 nM Reelin and/or synthetic Aβ42 (American Peptide Company Inc, Vista, CA, USA). After gently shaking for 20 min at 4 °C, the plate was incubated at 37 °C for 20 min. Then, cells were treated with 0.5 mg/ml Sulfo-NHS-SS biotin (Pierce) for 30 min at 4 °C to label surface proteins. Cells were lysed in PBS containing NaCl (150 mM), EDTA (5 mM), Nonidet P-40 (0.5% w/v), BSA (0.2%) and proteases inhibitors, and the biotinylated proteins were recovered with NeutrAvidin agarose (Pierce, Thermo Scientific; IL, USA) and analyzed in Western blots.

### Reelin and ApoER2 qRT-PCR analysis

RNA was extracted from human brains using the TRIzol^®^ Reagent in the PureLink™ Micro-to-Midi Total RNA Purification System (Life Technologies) following the manufacturer’s instructions. SuperScript™ III Reverse Transcriptase (Life Technologies) was used to synthesize cDNAs from this total RNA (2 μg) using random primers according to the manufacturer’s instructions. Quantitative PCR amplification was performed on a StepOne™ Real-Time PCR System (Applied Biosystems, Life Technologies) with TaqMan probes specific for human RELN (assay ID: HS01022646_m1 Applied Biosystems), human ApoER2 (assay ID: HS00182998_m1 Applied Biosystems) and human GAPDH as an endogenous controls (Applied Biosystems). The Reelin and ApoER2 transcripts were quantified using the relative standard curve method normalized to GAPDH from the same cDNA preparation, to confirm the specificity of the PCR products from the dissociation curves.

### Statistical analysis

All data were analyzed by one-way analysis of variance (ANOVA), or through a Student’s *t* test (two-tailed) for single pair-wise comparisons, determining the exact *p* values. When normality was rejected, a Mann-Whitney Rank Sum Test was used. The results are presented as the means ± SEM and all the analyses were all performed using SigmaStat (Version 2.0; SPSS Inc.). Correlations between variables were assessed by linear regression analyses. A *p* value  < 0.05 was considered significant.

## Additional Information

**How to cite this article**: Cuchillo-Ibañez, I. *et al.* The β-amyloid peptide compromises Reelin signaling in Alzheimer’s disease. *Sci. Rep.* **6**, 31646; doi: 10.1038/srep31646 (2016).

## Supplementary Material

Supplementary Information
