# Supplementary material for: The β-amyloid peptide compromises Reelin signaling in Alzheimer’s disease
Source: Sci Rep. 2016 Aug 17;6:31646. doi: 10.1038/srep31646 (PMC4987719; doi:10.1038/srep31646)
Supplement: Supplementary Information [file srep31646-s1.pdf]

The β-amyloid peptide compromises Reelin signaling in Alzheimer's disease

Abbreviated title: *Impaired Reelin signaling in AD*

Inmaculada Cuchillo-Ibañez[1,2,*], Trinidad Mata-Balaguer[1,2], Valeria Balmaceda[1,2,],

Juan José Arranz[3], Johannes Nimpf[4] and Javier Sáez-Valero[1,2,*]

[1]Instituto de Neurociencias de Alicante, Universidad Miguel Hernández-CSIC, Sant Joan d'Alacant, E-03550; [2]Centro de Investigación Biomédica en Red sobre Enfermedades Neurodegenerativas (CIBERNED), Spain; [3]Departamento Producción Animal, Universidad de León, León, Spain; [4]Department of Medical Biochemistry, Max F. Perutz Laboratories, Medical University of Vienna, 1030 Vienna, Austria.

Supplementary Figures:

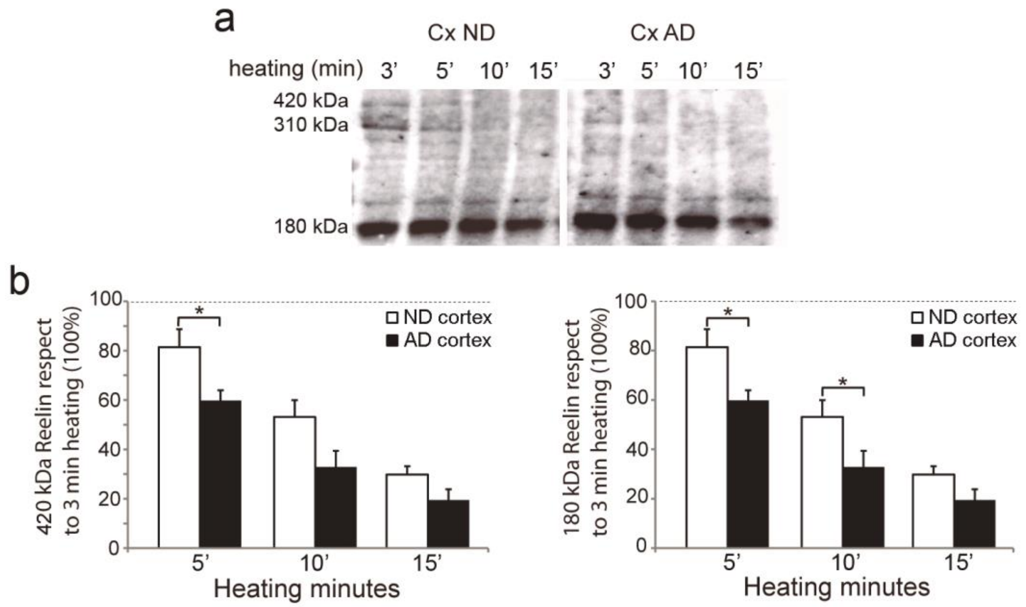

Supplementary Figure 1. Heating prior to electrophoresis affects the Reelin isolated from the non-disease (ND) and AD brain distinctly. In Western blots (**a**), we determined the effect of heating (time boiling to denature the samples for electrophoresis) on the estimation of soluble Reelin, comparing at 3 min heating in 5 ND and AD samples, the latter corresponding to advanced Braak stages (V to VI). Reelin extracted from AD brain displays differences in glycosylation pattern respect to ND, similarly as described in Fig. 5B. (**b**) The full-length 420 kDa and the major 180 kDa fragment were quantified. Each column represents the mean± SEM of each Reelin immunoreactive band normalized to the value estimated after 3 min heating (100%): *$p < 0.05$, **$p < 0.01$ using Student's *t*-test.

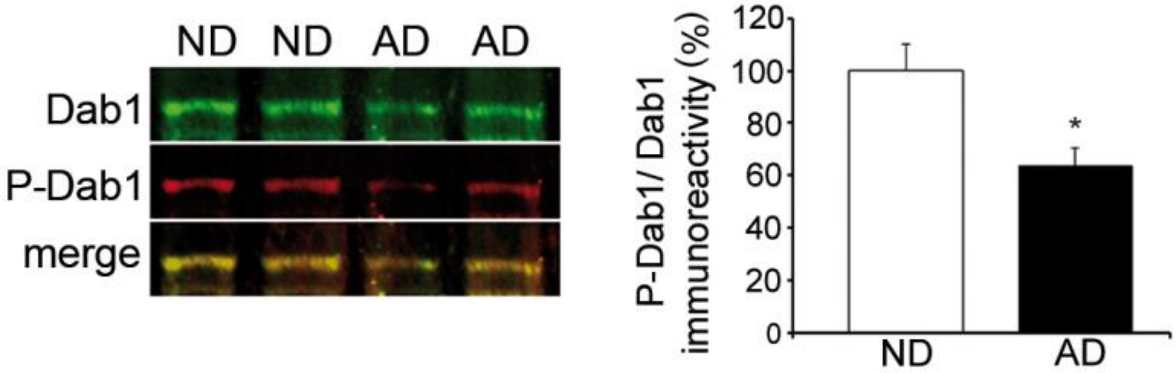

Supplementary Figure 2. Western blots of frontal cortex extracts from non-disease (ND; n= 12) and AD (n= 15) revealed by fluorescence simultaneously for the anti-Dab1 antibody (green) and an anti-phosphotyrosine Dab1 (red). Image showing co-localization (yellow) is also shown. Data represent the means± SEM of the ratios, normalized with respect to ND. *$p < 0.005$ using a Student's t test.

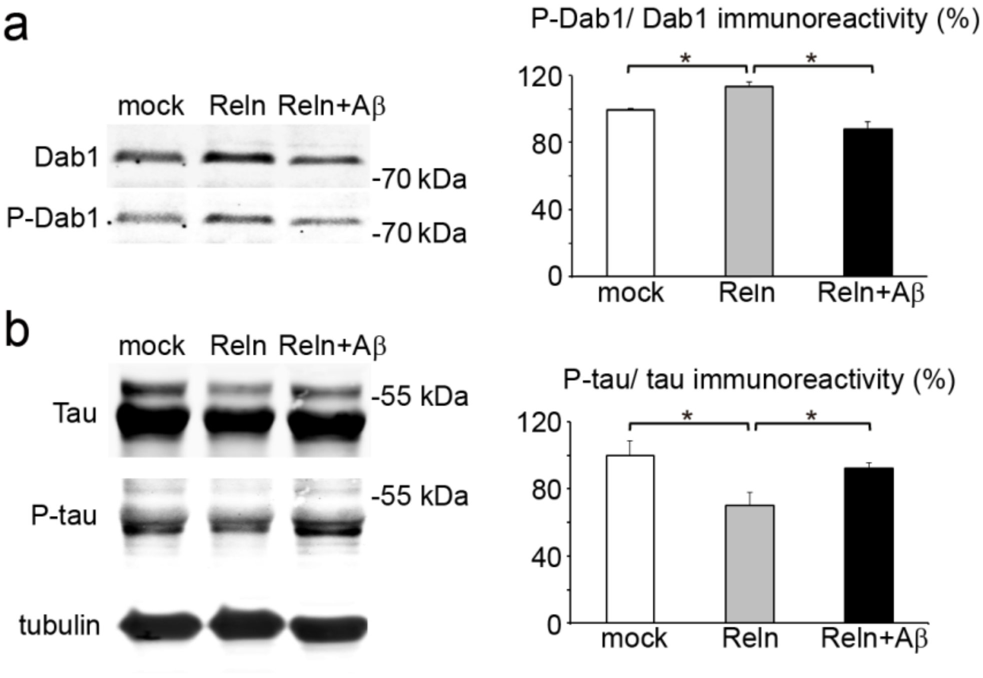

Supplementary Figure 3. (**a**) Levels of Dab1 and phosphotyrosine Y232 Dab1 (P-Dab1) were calculated in primary mouse cortical neuron cultures treated with similar amounts of Reelin (Reln; ~10 nM) or with Reelin previously incubated with Aβ42 at 2µM (Reln+Aβ). (**b**) After revealing for Dab1 and P-Dab1, neuronal extracts were also probed for total tau and phospho-tau (phospho Thr212+Ser212; AT100), and α-tubulin served as a loading control. The data represent the means± SEM (n= 6; from 2 independent experiments) of the ratio between immunoreactivity levels for the total protein and phosphorylated forms, normalized with respect to values obtained from neuronal extracts treated with mock. *$p < 0.05$ using a Student's *t* test.
